# A Blockchain-Based End-to-End Data Protection Model for Personal Health Records Sharing: A Fully Homomorphic Encryption Approach

**DOI:** 10.3390/s23010014

**Published:** 2022-12-20

**Authors:** Fausto Neri da Silva Vanin, Lucas Micol Policarpo, Rodrigo da Rosa Righi, Sandra Marlene Heck, Valter Ferreira da Silva, José Goldim, Cristiano André da Costa

**Affiliations:** 1 Applied Computing Graduate Program—PPGCA, Universidade do Vale do Rio dos Sinos (Unisinos) SOFTWARELAB, São Leopoldo 93022-000, Brazil; 2 Instituto Colaborativo de Blockchain—Instituto de Gestão Tecnológica e Inovação (ICOLAB), Porto Alegre 90540-010, Brazil; 3 Hospital de Clínicas de Porto Alegre, Porto Alegre 90035-903, Brazil

**Keywords:** health record, blockchain, encryption, distributed systems, medical informatics

## Abstract

Personal health records (PHR) represent health data managed by a specific individual. Traditional solutions rely on centralized architectures to store and distribute PHR, which are more vulnerable to security breaches. To address such problems, distributed network technologies, including blockchain and distributed hash tables (DHT) are used for processing, storing, and sharing health records. Furthermore, fully homomorphic encryption (FHE) is a set of techniques that allows the calculation of encrypted data, which can help to protect personal privacy in data sharing. In this context, we propose an architectural model that applies a DHT technique called the interplanetary protocol file system and blockchain networks to store and distribute data and metadata separately; two new elements, called data steward and shared data vault, are introduced in this regard. These new modules are responsible for segregating responsibilities from health institutions and promoting end-to-end encryption; therefore, a person can manage data encryption and requests for data sharing in addition to restricting access to data for a predefined period. In addition to supporting calculations on encrypted data, our contribution can be summarized as follows: (i) mitigation of risk to personal privacy by reducing the use of unencrypted data, and (ii) improvement of semantic interoperability among health institutions by using distributed networks for standardized PHR. We evaluated performance and storage occupation using a database with 1.3 million COVID-19 registries, which showed that combining FHE with distributed networks could redefine e-health paradigms.

## 1. Introduction

A personal health record (PHR) includes health information managed by an individual [1,2], while an electronic health record (EHR) is managed by the clinician and/or health-care institutions [3,4]. Personal privacy is a major concern in healthcare; therefore, in the context of PHR, individuals control the data and grant permission for access to third parties [5]. Healthcare institutions must comply with regulations, such as the Health Insurance Portability and Accountability Act (HIPAA) in the United States of America or the General Data Protection Policy Regulation (GDPR) in Europe [6]. Health institutions may face conflicts of interest when they, in addition to providing health care for patients, can also earn benefits, including financial gains, from data aggregation and provisioning [7,8,9].

In the past, technology players, such as Google and Microsoft, created platforms dedicated to PHR, where health institutions were able to share data with patients. Such solutions did not have much adoption, primarily because of a greater focus on EHR and a lack of integration with wearable devices and end-user health applications [10]. The centralized nature of their model also raised awareness regarding privacy and data usage, especially considering that such platforms offered other services for end-users and could potentially correlate health data with existing registries, which might result in undesired use of the health records [11].

In recent years, distributed network techniques for PHR have become relevant in the scientific community [12], focusing primarily on security and privacy [13], governance [14], and interoperability [4,15,16]. Such techniques can also be very helpful in emergency scenarios, such as the COVID-19 pandemic [17]. Most solutions rely on tamper-proof distributed ledgers, popularly known as blockchain [4,18,19]. Additionally, scalability is a challenge in blockchain solutions for PHR, and considering the vast amount of data, blockchain is not feasible in terms of computational resources and cost [19]. Thus, many studies consider storing only transaction history in the blockchain, while the PHR data are stored in off-chain infrastructures, such as cloud service providers or health institution premises [5,20,21,22]. Such centralized models expose personal data to undesired access and the infrastructure to cyberattacks that can cause data leaks [23,24,25]. In contrast, some solutions use a concept called distributed hash table (DHT), in which data portions are fractioned throughout participating sharding nodes, which can store bigger files more efficiently. The most popular DHT implementation is the interplanetary protocol file system (IPFS) [18,19,26].

This study proposes an architectural model for PHR end-to-end protections where data are decentralized in two formats: (1) PHR metadata (description and profile) stored on-chain and shared among all participants in the network and (2) PHR data (images, files) accessible off-chain through the IPFS network. Thus, individuals can use their own keys to provide data access to the PHR whenever requested by using end-to-end encryption and fully homomorphic encryption techniques (FHE) to support data analysis on the encrypted data. We introduce two new elements: (i) Data Steward (DS), which is a role in the network responsible for storing PHR on behalf of the individual, and (ii) Shared data vault (SDV), which is a temporary IPFS storage area where health institutions can access PHR with the consent of the individual. Both focus on segregating access to the PHR from individuals and providing control over the data. We evaluated the method using public-interest information regarding pandemics [27] and applied FHE to obtain statistical calculations on the encrypted data of a group of individuals. The results were obtained from a public dataset of 1.3 M cases from the US Centers for Disease Control and Prevention (CDC). We analyzed the algorithm performance, cryptography profiling, and security and privacy scenarios to identify the impact of the proposed model on each building block.

**Figure 1:**
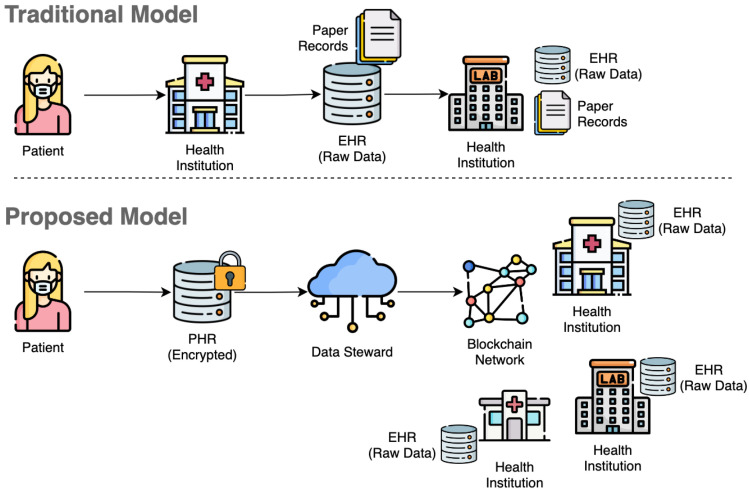
Solution contribution: In the traditional model for PHR management, the health institution is responsible for managing PHR data, and it is usually stored in unencrypted and paper formats. The proposed model provides control over PHR to individuals and promotes end-to-end encryption to protect privacy.

Our aim is to combine techniques that can elevate privacy protection and, at the same time, improve data access from health institutions, where the individual manages requests to PHR using a DS, which stores the PHR data encrypted with the individual’s key. We also aim to improve privacy in data analysis by adopting FHE techniques that allow health institutions to obtain numeric data analysis of encrypted data without exposing the individuals. The model promotes end-to-end encryption for data in transit and storage, which complies with regulations, such as HIPAA and GDPR, in the industry. Figure 1 illustrates the contributions of our study, highlighting the benefits of end-to-end encryption and a person-centered process. The main contributions of our study are as follows:

- Combination of IPFS and blockchain network to manage PHR data and metadata;
- Adoption of FHE techniques to reduce the demand for unencrypted data, supporting calculation on encrypted data;
- End-to-end encryption standardization to allow PHR data sharing and interoperability;
- Segregation of responsibilities regarding PHR to improve how individuals control personal data.

This paper is structured as follows. In Section 2, we describe the underlying concepts of the proposed model. Section 3 describes the related work and gaps in the literature. We present the proposed model in Section 4 and explore the development strategy in Section 5. In Section 5.4, we describe the evaluation criteria for the method and experiments. We present the results of the tests with a dataset on COVID-19 in Section 6 and discuss the results in Section 7. Potential limitations of the model are discussed in Section 8. The conclusions are presented in Section 9. The acronyms used in this article are summarized in Table 1.

## 2. Background

In this section, we cover the main aspects of our model regarding health, distributed networks (blockchain and IPFS), and Fully Homomorphic Encryption.

### 2.1. Blockchain

Blockchain technologies represent a unique design of ledger structure, distributed network, consensus protocol, and cryptographic mechanisms to promote transparency, data immutability, consistency, equal rights, and data availability [28]. Transactions trigger data processing and sharing in a blockchain. Transactions are grouped into blocks, and each block needs to meet specific consensus rules to be accepted by network peers [29]. Security in blockchain transactions is promoted by the use of cryptography algorithms and techniques, such as Asymmetric Key Pairs (such as RSA and Elliptic Curves), Hashing Algorithms, Hashing Hierarchy (such as Merkle and PATRICIA trees), Zero-Knowledge Proofs, and Homomorphic Encryption [30].

Networks are peered nodes exchanging data regarding transactions. Transaction finality may vary according to the adopted Consensus Algorithm, which is the method used by nodes to agree on each newly accepted block of transactions. Blockchain networks are usually divided into two major types: public and permissioned. In public networks, any computing node can participate with no need for permission of any participant besides the need to comply with the protocol (e.g., block size, block timing) agreed uponby existing members. Permissioned networks adopt governance protocols that are relied on outside the protocol itself. Thus, new nodes in the network need some approval to operate in the network. The concept of Blockchain Consortium emerges in this context, as consortia represent well how a permissioned blockchain network might work.

### 2.2. IPFS

Distributed Hash Tables (DHT) represent a content routing system in which data are distributed in a key-value format [31,32]. Unlike a Blockchain Distributed Ledger, a DHT shares Content Identifiers (CID) and peer lists that provide such files. There are multiple algorithms to manage DHT routing, including Content Access Network (CAN), Chord, Kademlia, Pastry, and Tapestry [4]. One of the most popular public DHT implementations is called Interplanetary File System (IPFS).

IPFS uses the Kademlia algorithm to manage its DHT [33]. Kademlia uses a binary tree to represent nodes with a given CID in the shortest path. Its protocol ensures each node knows at least one peer in its CID subtree if there is one. The Kademlia purpose is to build a DHT on top of three system parameters [34]:

1. An address space as a way that all of the network peers can be uniquely identified. In IPFS, this is all the numbers from 0 to $2^{256}-1$;
2. A metric to order the peers in the address space and, therefore, visualize all the peers along a line ordered from smallest to largest. IPFS takes SHA256(PeerID) and interprets it as an integer between 0 and $2^{256}-1$;
3. A projection that will take a record key and calculate a position in the address space where the peer or peers most ideally suited to store the record should be near. IPFS uses SHA256(RecordKey).

### 2.3. Fully Homomorphic Encryption

A homomorphic cryptosystem on a given message space *M* is a quadruple (K,E,D,A) of probabilistically expected time-based algorithms conforming to the following conditions [35]:

- Key Generation (K): a key pair ($k_{e},k_{d}$)=k∈K where *K* represents the key space. Calculation algorithms are highly dependent on the *K* element;
- Encryption (E): consists of applying key $k_{e}$ on a message m∈M, producing a ciphertext *c* in the cipher-space *C* where c∈C;
- Decryption (D): consists of applying the key $k_{d}$ on an encrypted message *c* to produce m∈M; Homomorphic Property (A): is a scheme that requires $c_{1},c_{2}\in C$ to produce a third element $c_{3}\in C$ such that $\forall m_{1},m_{2}\in M$ holds only when $m_{3}=m_{1}{\dot{m}}_{2}$.

Homomorphic Encryption could support mathematical operations, such as summation, multiplication, and logic XOR operation [36]. Techniques that support only one type of operation (summation or multiplication) are called Partially Homomorphic Encryption. On the other hand, systems that support both calculations are called Fully Homomorphic Encryption (FHE) systems.

## 3. Related Work

Before developing the proposed model, we revised relevant studies in the field of Computer Science and Bioinformatics that used distributed networks and cryptography to protect personal privacy, and support interoperability in the context of PHR. We analyzed the studies based on the following criteria:

- Level of control over data (create, share, revoke, delete);
- Location of the PHR data (on-chain/off-chain);
- Data protection in each step (encryption, vulnerabilities).

Regarding the control individuals have over data, Table 2 compares related studies considering the relevant aspects of how users manage data and operations. Most studies provide ways for individuals to formally grant access to the PHR data. However, some models assume that whenever individuals participate in the model, access to data is automatically granted [4,20,21,26] without further authorization.

When a third party generates an individuals’ private key, privacy issues may occur, which is why encryption key pair generation is a crucial step, and a few models consider the individuals to be responsible for generating their own keys [4,19,41,42,43,44]. Most models do not provide ways for users to formally revoke access once granted, with the notable exceptions of the models by Sonkamble et al. [37], Misbhauddin et al. [19], and Ghani et al. [43]. A few models allow users to encrypt PHR data [4,21,42,44], and only the OmniPHR model of Roehrs et al. [4] and the model designed by Wang et al. [44] provide methods for users to manage data locations; most related studies only consider health institutions responsible for managing data location.

We divided the data location into three groups: centralized location (on-premises and cloud service provider), decentralized file solution (IPFS and DHT), and inside participating nodes in a decentralized network. The first two are considered off-chain storage, whereas the third is considered on-chain storage. Most conventional models combine on-chain with off-chain storage because storing all medical records on-chain can raise scalability issues, as data are replicated in most or all nodes in the network.

Most models use only the hash of PHR data on-chain [19,21,22,26,40,42], while the MyBlockEHR model of Sonkamble et al. [37] placed lightweight data on-chain and bigger data files, such as scan images and medical reports, off-chain. The model developed by Mahdy [41] considered medical records to be on-chain, whereas most models consider medical records to be off-chain. Some studies have adopted decentralized file storage, such as IPFS or DHT [4,18,19,26], as a solution for scalability. The data locations used in previous studies are summarized in Table 3.

Considering data protection, we focus on analyzing the closeness of each previously reported model to an end-to-end encryption model. We consider three major events in the health data lifecycle: (1) data origination (by the user or health institution), (2) data distribution (interoperability and sharing), and (3) data retirement. Most studies considered data origination only in the context of medical institutions. However, some models consider users to have their nodes in a decentralized network (via a computer, smartphone, or IoT device).

Regarding data access, some models assume that whenever a person participates in a model, access to data is automatically granted [4,20,21,26]. Some models apply encryption to PHR using specific role keys (doctor, patient, and nurse) [4,19,42], while others share data in an unencrypted format [20,37,38,41]. A few models allow individuals to encrypt PHR data [4,21,42,44]. There is an opportunity to elevate the security level and control of the PHR lifecycle from its origination to how it interoperates among health institutions.

Some models execute data distribution by applying encryption using each specific actor role (doctor, patient, and nurse) [4,19,42], while others share data in a plain format [20,37,38,41]. Madine et al. [42] applied proxy re-encryption to guarantee plain text access only to authorized requesters. Ghadamyari and Shame [20] and She et al. [21] utilized homomorphic encryption to support encrypted data calculation to protect personal privacy. No module provides a means for data retirement despite regulations, including HIPAA and GDPR, recommending such alternatives.

## 4. Proposed Model

**Figure 2:**
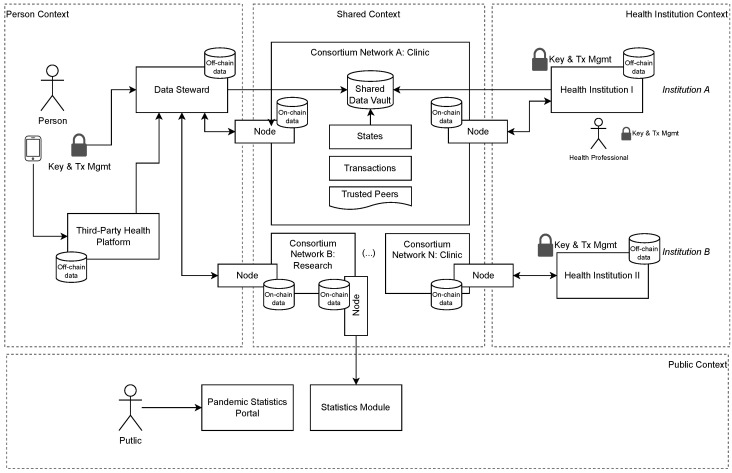
Proposed solution organized in multiple contexts: Personal context for data collected outside the medical environment, health institution context for data collected in a medical environment, the public context for statistical calculation and visualization of FHE, and shared context for the exchange of data among all other contexts.

This study proposes a model to address two significant issues regarding PHR interoperability in distributed health systems: ensuring personal control over data and obtaining relevant information without exposing any individual data. The proposed method combines blockchain architecture with IPFS and FHE to deliver a distributed health system in which users control their data and support relevant information calculations of the encrypted data. An overview of the proposed model is provided in Figure 2.

This study is an extension of our previous study [4], in which a model using DHT networks is proposed to store PHR. The previous model does not use blockchain networks or FHE techniques. There was no formal authorization for PHR consumption; therefore, whenever a person participated in the network, all data became immediately available. In the proposed model, we address all these issues with a structure organized in multiple contexts.

1. The personal context relates all data collected by electronic devices, applications, and third-party health platforms for an individual. Each individual has their own key pair to encrypt and send the data to a DS;
2. The health institution context is where a given health institution keeps all records that originated in their own premises or were obtained from persons or peer institutions in a consortium. Each institution keeps a list of peers in the consortium and can share data with other peer institutions, using their own key pair to sign transactions;
3. The public context contains elements that pull encrypted data from the network and performs FHE calculations to generate data analysis (statistics module) and can publish the results in a public portal;
4. The shared context is dedicated to exchanging data from personal and health institution contexts using the distributed network for data (IPFS) and metadata (blockchain).

**Figure 3:**
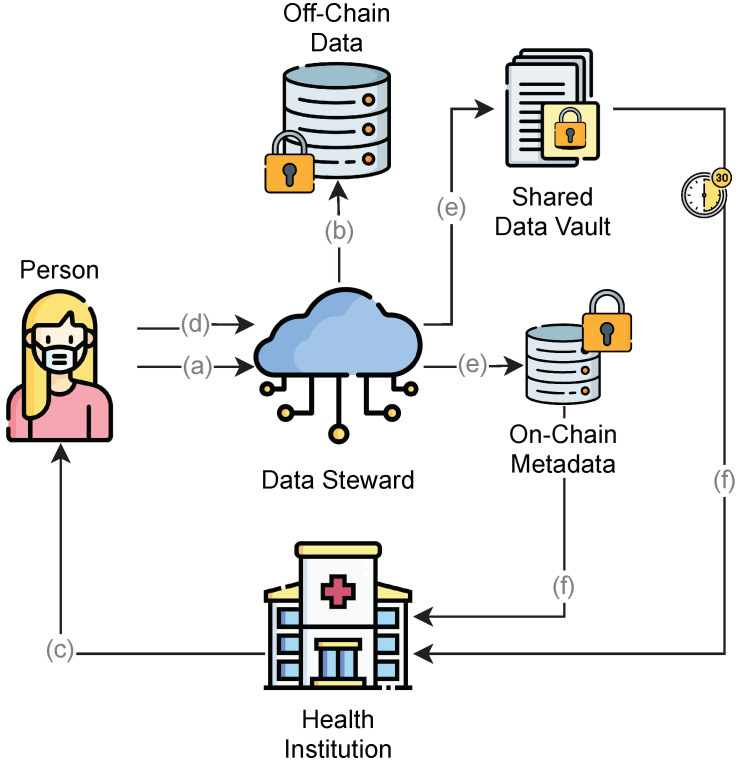
Data flow of the solution: The user sends authorization to the DS for a given consortium. Health institutions can send the data back to the DSs as well.

The model flow is shown in Figure 3. Our focus is on ensuring personal control over PHR data throughout the entire lifecycle, applying end-to-end encryption to improve privacy, separating data from metadata to improve scalability, using role segregation to prevent conflicts of interest, and applying FHE to provide flexibility with calculations, even on encrypted data. We introduce the role of a DS, which runs IPFS nodes to manage PHR data via temporary areas called data vaults, triggered by the requesters and approved by the users. To develop our proposed model, we take into consideration the following design principles:

1. The user or any caregiver/authorized personnel has control of all data access.
2. End-to-end encryption, even during processing.
3. Use of on-chain storage for metadata and off-chain (IPFS) storage for PHR data.
4. Any authorization to data access must have a predefined time frame.
5. Access revoking and data retirement.

### 4.1. Person-Centered Data Flow and Responsibility Segregation

Personal control over PHR is an important characteristic of the proposed model. We define control in the context of our model as the ability to establish an authoritative data source that aggregates data from multiple origins and is encrypted and managed by the corresponding user. Health institutions can generate a series of registries concerning an individual. In such scenarios, the corresponding individual may have access to such a PHR and replicate it in infrastructure under their management.

One of our design principles is “the user (or any caregiver/authorized personnel) controls all data access”. To accomplish that, we assume that the individuals manage their own cryptographic key pairs using a solution, such as a mobile application or specific hardware, that can hold the private and public keys for the user and digitally sign transactions. The private key encrypts data and signs transactions, and the health institution uses the public key to encrypt personal data. Individuals can store data in many different ways, on their premises or in a specialized service provider, such as a DS.

We take into consideration two different scenarios where health institutions may request data from an individual, as described below:

1. Data from user to health institution: Each individual manages their information, and any access to it must be formally approved. This approach complies with existing regulations, including GDPR and HIPAA;
2. Data from one health institution to another: We consider in our model that permission is conceded at a consortium level, which implies that whenever an individual allows access to their data, all health institutions participating in the same consortium have access to such data. It indicates that, in this specific scenario, if the individual has already given access to their health data, the requesting health institution can query the blockchain for existing SDVs to fulfill their needs.

The proposed model considers health institutions as consortium members for specific purposes (e.g., clinic, research, and data analysis). Whenever a health institution generates data on a particular individual (a), it must be provided to the corresponding individual to store and protect new records. In every circumstance in which demand for personal data exists (b), the individual (or a previously authorized person) formally grants access to the requested data for a predefined period and specific consumer (c).

All requested data are provided to a specific health consortium encrypted with the public key of the requesting institution (d). The requesting institution can fetch data, decrypt it outside the blockchain, and provide the data to other consortium members whenever requested. Individuals have the right to revoke access to the data or retire the data at any time.

Each piece of data stored outside any shared storage mechanism is considered to be off-chain, while data already shared among the participants in a distributed network are considered to be on-chain. Our model considers the metadata to be on-chain and the PHR to be off-chain. The access to data is delegated from the individual to the DS and from the DS to the health institutions in the consortium to respect the design principles and to guarantee end-to-end encryption and control over data access, scope, and availability for a predefined period.

We consider a scenario in which multiple health institutions organize themselves as a consortium with a body of governance and mutual rules on interoperability, data coding, and purpose regarding shared health records. Consortium members provide nodes in the distributed network to submit and validate transactions and synchronize data states that are made accessible for interoperability. Each participating institution remains individually subject to existing laws and regulations, and we assume that all operations regarding the data of a specific person occur under their consent and the applicable regulation. Consortium members are also responsible for guaranteeing that they use data only for the requested purpose.

### 4.2. Data Steward

DS plays a role in our model, which focuses on storing personal data separate from health institutions. This separation aims to prevent conflicts of interest on whether health institutions benefit from data (marketing, pricing, trials, and research) and provide healthcare services simultaneously. It acts as a service provider, and the role can be performed by public or private institutions. Each data steward represents a distributed network of IPFS/blockchain nodes that store data on behalf of the individuals, originating from third-party health solutions, such as sensors, monitoring devices, or mobile applications, and encrypted using the corresponding personal public key.

Health data are made available in an SDV whenever requested and approved by the individual. The requests are data payloads in the following format: *{timestamp, consortium-public-key, institution-public-key, requester-public-key, data-scope, finality, requested-period}*. The payload may not contain any sensitive data from individuals and/or institutions and can be in the form of a QR code, accessible by individuals through a mobile application with their private key to sign the payload and send a transaction to the DS authorizing data access for a period. Requested data are re-encrypted by individuals using the requester’s public key and posted to a shared data vault.

Individuals can permit a specific health institution or health consortium. When allowing access to a consortium, certain techniques, such as attribute-based encryption [39], can be adopted to provide access to all members of the consortium. Whenever a person authorizes access to a set of PHR, the consumers (individual institution or consortium) must be informed, and the purpose and period must be explicitly mentioned. Individuals and their data are protected by the regulation, and the proposed model provides support for all regulatory requirements.

### 4.3. Shared Data Vault

SDVs represent temporary file content identification. (CID), a unique hash identification used to retrieve data distributed on the IPFS network and made available to fulfill a specific request from a health institution. They are created by a DS, only with express authorization from the individual, for a predefined purpose and period using encrypted data sent directly from them. For interoperability, shared data should respect the following conditions:

- Personal identifiers: Each individual has their own key pair that works as an identification towards other participants in the system. As our model focuses on preserving personal privacy, anonymization and encryption mechanisms are taken into consideration;
- Data format: Based on the literature, we consider data and metadata to be stored separately (on-chain and off-chain, respectively). All data in the standardized formats [45] (HL7 FHIR [46] and OpenEHR [47]) are stored off-chain in an IPFS network;
- Data coding: The separation between the data and metadata allows health institutions to access data descriptions on-chain and then request access to specific data based on the metadata via a person or other health institution in the same consortium. Data are made available in an SDV in a standardized format. To support FHE calculations, data in an encrypted numeric format is included in the provided data.

In those scenarios where health institutions request PHR from an individual, they consume data from an SDV to fulfill a request. Whenever data originate at health institutions, such institutions never act as consumers, considering that the data are already under its management and no further authorization is needed. Whenever a portion of the data is made available in the SDV, a transaction containing such data is sent to the blockchain. Health institutions participating in the consortium can read the blockchain for the public key of a specific individual and data purpose to receive all the information that leads to the corresponding SDV.

To meet the design guidelines of “all data access will have a predefined time frame”, each data vault should have a predefined expiration time. The combination of different elements accomplishes data expiration:

- The SDV is a temporary data area available via a computational unit managed by a DS. Each SDV is a file registry in an IPFS cluster, with a unique content identifier CID shared by a process called pinning. After expiration, peer nodes must run a process called unpinning (cease API availability) and garbage collector (remove local unpinned files). It might be accomplished using solutions, such as the IPFS cluster (Information on IPFS cluster is available at https://ipfscluster.io/—accessed on 15 October 2022);
- The communication between a data requester and DS regarding a specific SDV uses JSON Web Token (JWT) (More information on JWT specifications is available at https://datatracker.ietf.org/doc/html/rfc7519—accessed on 15 October 2022, a web standard for communication between two parties with support to key expiration). After a JWT token expires, access to a specific SDV is denied;
- The period of data availability is proposed by the requester and accepted by the individual. After data expiration, requesters must submit a signed transaction to the blockchain network regarding the removal of any copy of the corresponding SDV. The signed message must contain useful information, such as the file CID, to allow further auditing;
- Health institutions may keep medical records necessary for further procedures and covered by regulations, such as HIPAA or GDPR. The final transaction is a signal to the participants in the network that the corresponding SDV is no longer necessary and can be removed.

**Figure 4:**
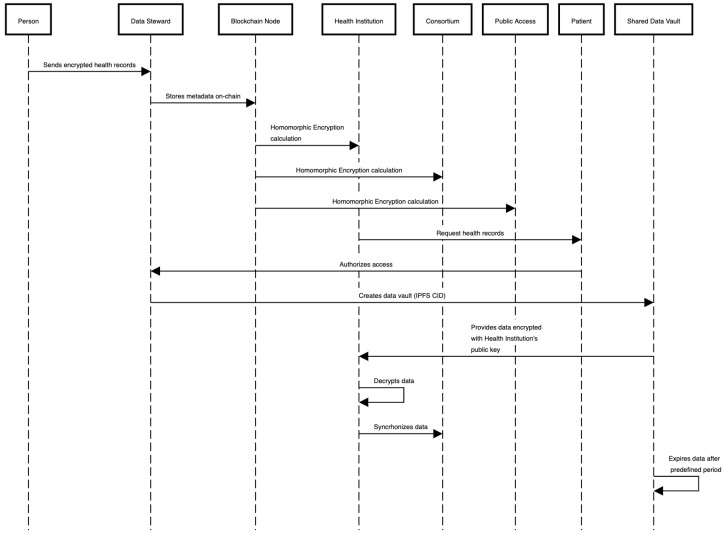
Solution sequence diagram with the main causes of use: (1) Individual shares data with the IPFS network and metadata with the blockchain nodes; (2) Individual authorizes the health institution and consortium to access a portion of data in an SDV for a predefined period.

FHE allows the system to execute mathematical operations on the encrypted data. Therefore, all data should be in the numeric array format for the FHE calculations. In the case of raw data, access management should be presented in the form of a sequence diagram, as shown in Figure 4:

1. The individual authorizes a given consortium to access their data.
2. The individual requires the creation of a data vault and data scope from the DS.
3. The individual decrypts the data with a private key and encrypts it with the public key of the health institution.
4. The data steward creates an SDV and returns the CID to the individual.
5. The individual shares the CID with the health institution.
6. Once the time window expires, the DS removes (unpin) the file from the IPFS network.

As SDVs store data encrypted with the public key of a specific institution, the given institution is able to decrypt the data using its private key. This situation does not represent any privacy exposition security issues because only individuals can authorize such access for a predefined period.

## 5. Methodology Development

To evaluate the proposed model, we developed a methodology that is applied using techniques developed previously [20,21,29] that focused on PHR interoperability and data sharing. Our results complement the results obtained in previous studies and address the literature gaps related to data protection and network scalability. The methodology consists of the following steps:

1. Design new architectural elements to segregate the responsibility for the PHR.
2. Evaluate FHE techniques to promote end-to-end encryption and to support calculations of the encrypted data.
3. Evaluate technical aspects with respect to processing time, scalability, and storage occupation on distributed networks.
4. Analyze risk scenarios related to data security and personal privacy.

This section presents the criteria of the proposed model and describes how computing occurs in a distributed network and how to perform statistical calculations on the encrypted data. A practical example demonstrates how each building block of the model behaves in each circumstance. The implementation of the model considers the cases described in Table 4.

### 5.1. End-to-End Data Protection

PHR sharing involves a series of players storing and exchanging data. The proposed model introduces the role of a DS to maintain data for a person and support sharing with the health institutions. DSs are ’trustless’ platforms, as it is not necessary to trust DS behavior when using their services. To accomplish this, the proposed model uses a series of cryptography mechanisms to protect data and privacy.

Cryptography key management systems support multiple alternatives to elevate security or improve the user experience while managing cryptography keys and transaction signing. Here, we consider single- and multi-signature keys as possible solutions. In terms of key management, there are solutions for end-users, usually called cryptographic wallets (For more about cryptographic wallets, please refer to this post at coinbase.com: https://www.coinbase.com/learn/crypto-basics/what-is-a-crypto-wallet—accessed on 15 October 2022. Such wallets can be in the form of physical devices (paper, smart card, and hardware) or digital applications, such as mobile applications and web browser extensions. Cryptographic wallets usually offer functionalities to handle the following steps in the key lifecycle:)

1. Key generation: Process to determine whether the private key is created. The owner must keep their private keys safe to be able to recover them whenever required;
2. Digital Signature: Using a private key, the owner can generate an encrypted version of the input data. We consider two types of data to be signed by owners: PHR and blockchain transactions;
3. Recover: Based on a previously saved private key, owners can recreate their keys and transactions in a new key manager whenever necessary, as in the setup of a new mobile device.

All data signed with a personal private key allow for the identification of the corresponding public key. Thus, all the events triggered by a particular individual are verified. All encrypted PHR should be stored in a specific database, apart from transactions, referring to their corresponding transactions.

**Figure 5:**
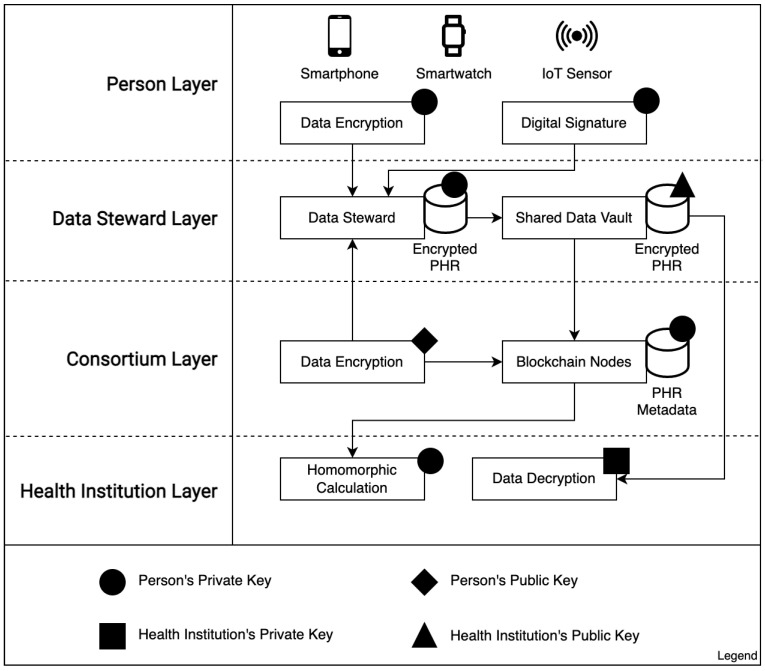
Key management and end-to-end encryption: Throughout their lifecycle, PHRs rely on different layers and demand proper encryption/decryption processes to fulfill requests from health institutions and private individuals.

Data sharing should create two elements: the SDV on the IPFS network and a transaction in the blockchain referring to the vault (web address, content description, expiration time, and public key of the individual). Thus, we may have efficient storage management, as each consumer can choose to store only transactions and retrieve corresponding data on demand. This enables individuals to request the removal of a specific amount of data from the network without the need to remove its corresponding transaction. In Figure 5, we present the application of the encryption and decryption keys in each layer of our model.

Each layer in Figure 5 represents a specific context where data are generated and/or stored. In the person layer, individuals use their private keys to generate data, share it with a DS network (data encryption), and manage incoming requests for the data access (data signatures). At the DS layer, the IPFS-distributed network keeps copies of health records encoded with the private key of the individual. Whenever a person requests, SDVs with data encoded are set up with temporary access using the public key of the health institution. At the consortium layer, blockchain nodes keep metadata records encrypted with the private key of the individual and generate new health records encoded with the public key of the individual to share with them. At the health institution layer, encrypted metadata from the blockchain nodes can support homomorphic calculations, and incoming data from SDVs can be decoded using the private key of the health institution.

### 5.2. Distributed Network

Data consolidation is conducted at the network level using distributed ledger technology (blockchain). Each participant in the network is provided a dedicated node. Each node reaches a consensus on transactions with peer consortium members and maintains an updated version of the ledger. Submission of the following types of transactions is expected in the network:

- PHR signed by an individual;
- PHR signed by a medical institution;
- A new SDV signed by an individual;
- Data expiration signed by an individual.

Data validation and processing occur at two complementary levels: The *transaction level* is responsible for composing blocks of transactions to ensure encryption validity (e.g., transaction sender and receiver, block hash). The *health record level* is responsible for verifying incoming transactions in terms of transaction sender roles and data signatures and for performing homomorphic calculations to analyze data validity (e.g., the body temperature should range between an upper and lower level).

Data at both levels are encrypted; therefore, it is impossible to perform validation using plain data. Thus, validating nodes must add a *trust weight* to the incoming transactions in a block and elevate trust and mitigate unexpected behavior in the network. Validating nodes can also refuse to accept incoming transactions based on their specific rules.

### 5.3. Statistical Calculation on Encrypted Data

In the public context, information is available to the public via elements, such as a statistical portal, as proposed in this model. A statistical module processes all calculations regarding data in the distributed network and conducts homomorphic calculations to obtain meaningful information.

The statistical module executes this process in two steps: sampling and statistical calculations. The list of trusted peers can influence the data selection criteria in the sampling process. The resulting calculations can be shared on web portals because this information is of public interest.

### 5.4. Evaluation Criteria

Performance benchmark tests run data processing on encrypted data and compare the results to that of plain data processing. It must be considered that such calculations process millions of records in a matter of hours, even for encrypted data. Some technical aspects are relevant for a detailed evaluation of the proposed model, as described below:

- Benchmark of the calculation process on the encrypted and non-encrypted data;
- Data encryption, decryption, and calculation profiling;
- Storage occupation for plain text, encrypted, and encrypted-compressed data formats;
- Block propagation time for different data formats;
- Security aspects for key management and data lifecycle.

We selected an open dataset from the CDC (The dataset is available on the CDC website: https://data.cdc.gov/Case-Surveillance/COVID-19-Case-Surveillance-Public-Use-Data/vbim-akqf—accessed on 15 October 2022) [48]. The dataset includes 22.5 million records of anonymized patient data. We chose a subset of data related to people aged 60–69 years, which resulted in a total of 1.285 million records.

For the development of the model, we use the SEAL library [49], which implements the Brakerski, Fan, and Vercauteren (BFV) algorithms for FHE [50]. The application code is written in JavaScript, with the Node.js version of the SEAL Library, and data are loaded from CSV files in plain text and encrypted during execution. The application simulates a blockchain node that calculates the number of infected patients in the dataset. For data compression, the standard *zlib* package from Node.js was used. To calculate block propagation time, we used a blockchain network simulation tool called SimBlock [51].

We evaluated the processing time performance on the encrypted data, and compared it with plain data processing, as described in a previous study [52], by organizing the data into groups of 100,000, 300,000, 600,000, and 1.2 million records. The encryption process analyzes different key sizes for polynomial modulus n∈{1024,2048,4096}, which is equivalent to that of a previously established method [20,29], while security and privacy scenarios are analyzed with a set of experimental scenarios, as in a previous study [21]. The network simulation is based on a previously developed simulation process [21] and simulates a network with three different regions running 10, 30, and 100 nodes, with block sizes varying from 535 to 2140 KB. In addition, all experiments were run on a 3.2 MHz, 8-core computer with 16 GB RAM.

## 6. Results

This section presents and discusses the experimental results, focusing on the algorithm performance, profiling and data privacy, and security scenarios. We summarize the main results in Table 5.

### 6.1. Performance Evaluation

In this subsection, we analyze the performance of the FHE algorithm in the context of the proposed model. We aim to measure the time required to encrypt, process, and decrypt the PHR data. Thus, we set up a scenario in which a node in the network must be calculated over an entire set of encrypted data.

The BFV settings of the FHE algorithm are as follows: degree of the polynomial modulus n=4096 and coefficient modulus q=109 (according to the default recommendation in [49]) with a 128-bit security level. The results of comparing plain text and encrypted data processing show that for more than one million records, computation on the encrypted data is executed in less than 3 min, even when processed by regular hardware.

Kocabas et al. [29] processed a group of 440 registries in an estimated time of 70 ms. If we propagate this performance to 100,000 registries, which is our minimum dataset size, we arrive at an estimated time of 15.9 s, which is higher than our result by 42.6%.

We divided the FHE processing down into three major steps: encryption, decryption, and calculation, and varied the polynomial modulus n∈{1024,2048,4096} with the corresponding coefficient modulus q∈{27,52,86}; the parameter setting was based on a previous study [29,36]. The results, as presented in Table 6, demonstrate that the encryption process consumes approximately 48% of the processing time, decryption consumes 52% of the processing time, and the additional calculation consumes less than 1% of the total time.

In comparison to the results obtained by Kocabas et al. [29], our results achieve an overall better performance with respect to FHE calculation steps. The execution of the encryption takes took approximately 1.65 s in the method proposed by Kocabas et al., whereas our tests took ∼2.4 ms with higher (n,q) parameters. In the aforementioned study, decryption was achieved in approximately 650 ms, while our model achieved decryption in ∼2.6 ms. Further, in the aforementioned study, addition was accomplished in 0.11 ms, while it was achieved in ∼0.01 ms in our experiments.

**Figure 6:**
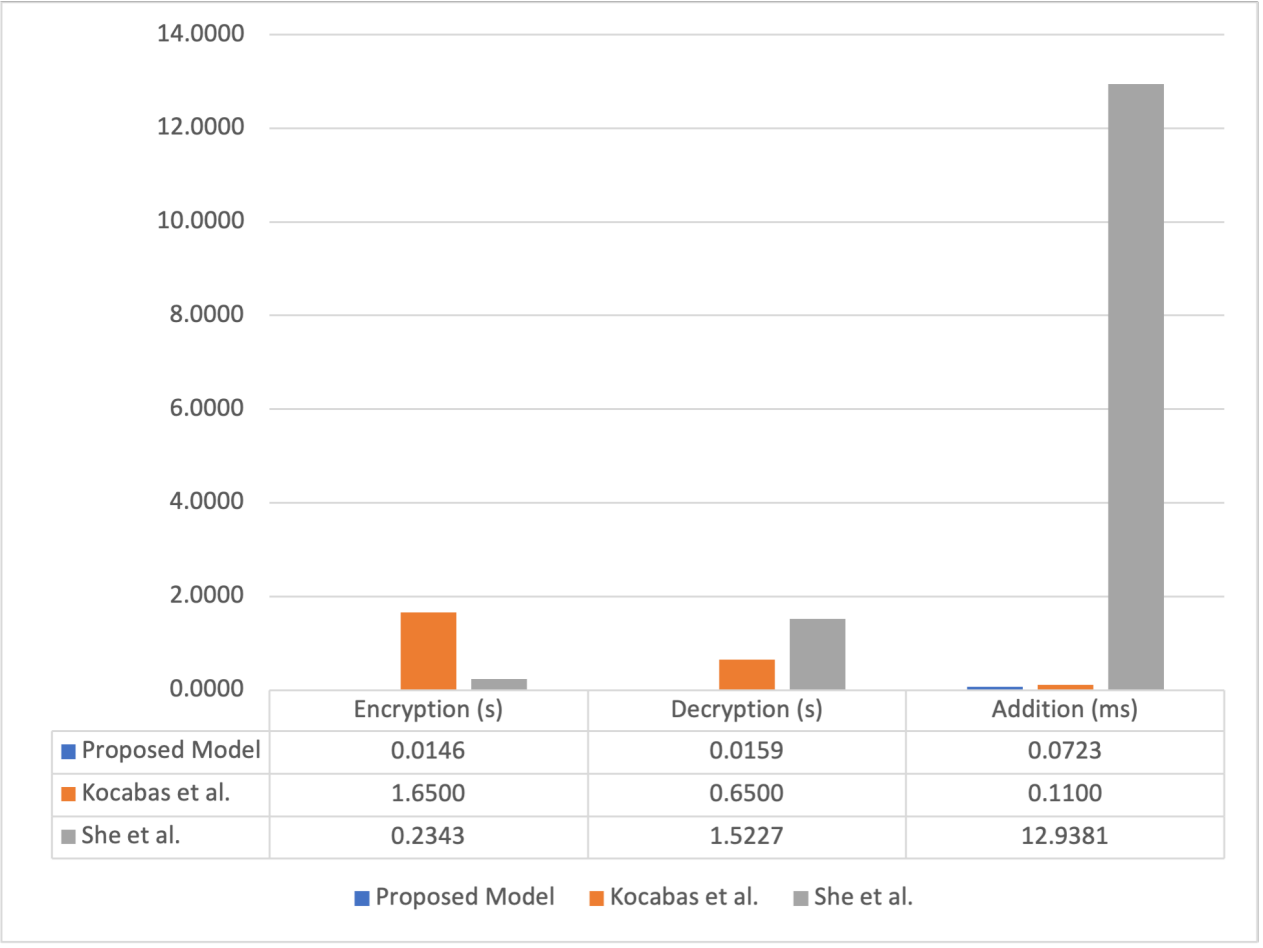
Benchmark profiling: the proposed model has a faster processing time when compared to related work of [21,53]. As we can see in the right column, She et al. used the Paillier algorithm to reach a 128-bit security engine, which demands a prohibitive processing time.

When compared to [21], the proposed model can support addition and multiplication operations on encrypted data by using of FHE algorithm (BFV) instead of Partially Homomorphic Encryption (Paillier). BFV also has better performance when compared to Paillier. We considered a 128-bit security level parameter, as stated in [54], to compare both aforesaid algorithms, then we extrapolated it to our database to obtain a metric and compare the models. We show the result in Figure 6.

We analyzed the storage consumption by considering parameters *n* and *q* for the polynomial modulus degree and coefficient modulus, respectively, in the BFV encryption based on previously reported results [36]. For each (n,q) pair, we measured the string size in plain text, encrypted data, and compressed encrypted data. For compression in Node.js, we used the library *zlib*. Each test round included 100,000 registries. The results are presented in Table 7.

The total space required to store 100,000 registries in the encrypted format can reach approximately 7 GB of storage with a higher (n,q) encryption level. We reached a compression rate of ≈24%, which could reduce the storage space to approximately 1.7 GB. In the study conducted by Kocabas et al. [29], each registry consumed approximately 65 KB, which was approximately 20% more than the compressed average for n=4096.

### 6.2. Block Propagation Time

To estimate the time required to propagate a given block pool size to a blockchain network, we simulated a network using SimBlock [51]. The network parameters are as follows: three regions (North America, Europe, and South America), block time of 5 s, blockchain header size of 500 bytes, and average pool size of 10,000 registries.

**Figure 7:**
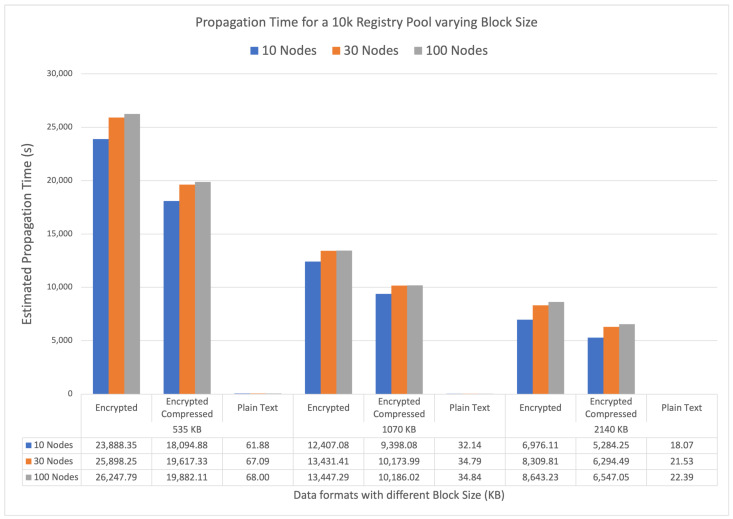
Blockchain network simulation: 10,000 pool size with block header of 500 B and block time of 5 s. Different scenarios compare different block sizes (535, 1070, and 2140 KB) in three formats (plain text, encrypted cyphertext, and compressed and encrypted cyphertext) with 10, 30, and 100 nodes distributed in three different regions. The chart depicts the time in seconds for 10,000 registries to propagate in the network.

We tested the network by varying the following parameters: the number of nodes in the network n∈{10,30,100}, the block size (in bytes) b∈{535,1070,2140}, and the median record size for n=4096 encryption with 212 B for plain text, 84,924 B for encrypted cyphertext, and 64,328 B for compressed and encrypted cyphertext. Each simulation round generated 100 blocks with the corresponding propagation time in seconds. Figure 7 presents the results for each scenario.

Our network is based on the work of She et al. [21]; however, as the aforementioned study does not provide information on the propagation time, it is not possible to compare the results. Nevertheless, for plain text data, propagation is achieved in a few seconds, while encrypted and compressed encrypted cyphertexts in 10,000 registries are propagated in hours. Larger block sizes provide a more efficient propagation time, and compressed cyphertext provides a 20% to 25% time reduction in block propagation.

### 6.3. Security and Privacy Analysis

Data security and personal privacy are critical aspects of PHR interoperability. Based on a previous study [21,22], we propose a set of vulnerability scenarios to analyze how each scenario affects each model’s building blocks from a security and privacy standpoint.

1. Personal privacy is compromised when someone obtains unauthorized access to the personal key $k_{e}$ or to any other means of communication with the DS or any third-party health platform (off-chain data). Considering all PHR are encrypted with the personal key, requests for SDV must provide a public key. An individual does not have access to another individual’s data. In this case, DSs can implement two-factor authentication to mitigate access to sensitive data, and requesters can demand the participation of trusted peers in order to mitigate unauthorized access;
2. The security of a DS is compromised when the off-chain records or the private key $k_{e}$ of the DS allows unauthorized access. Considering all PHRs are encrypted with the personal key $k_{e}$, DSs cannot create an SDV without authorization (signed transaction). In this case, there is no PHR exposition as they are encrypted, and DSs do not have access to the individuals’ private key;
3. The private key of a medical institution is compromised when unauthorized access to key $k_{e}$ requests DSs for a data vault. Considering all PHRs are encrypted by each corresponding personal key $k_{e}$, DSs cannot create an SDV without the authorization of the individual (signed transaction). Any open SDV has a limited duration. In this case, only data in open SDVs are subject to exposition for some time;
4. A node in the consortium is compromised when the server running the node or the private key suffers unauthorized access. Considering nodes receive transactions with encrypted data, their corresponding counterpart signs each transaction. Each node synchronizes blocks with other peers with the encrypted data and with peer nodes in a consortium. In this case. all data available to the compromised node in the data vaults become accessible for a while; peer nodes in the consortium can ban the compromised node from the network and remove access to the data.

Table 8 presents an analysis of the above-mentioned scenarios. Most of the risks associated with the proposed model rely on steps before sharing with the distributed network, as private keys of users or data access can target unauthorized personnel. When compared to [44], our work has a higher security level by the use of asymmetric keys instead of symmetric keys.

When compared to the work of [43], our model has a more secure approach by the use of end-to-end encryption, while the related work relied on steganography to protect data using Shamirś Secret Sharing algorithm [55]. Such a scheme encodes a secret *S* as the constant term of a polynomial f(x) of degree t−1 defined over a fixed finite field *k*. A share of the secret is a pair $\left(xi,f\left(xi\right)\right)\in k^{2}$. It demands a number of *t* different nodes to store shares and reconstruct the secret, while in the proposed model, any number of *t* is sufficient, as data are encoded with the requesterś public key instead of the steward’s public key, as in [43].

## 7. Discussion

The proposed model proves that it is possible to process the new PHR of an individual in less than 1 s to fully encrypt it, thereby consuming less than 10 KB of storage. This is computed by a DS and shared with a blockchain consortium with 100 institutions across three different continents in less than 30 s. It is a significant benefit to the segment because it protects individual records and simultaneously supports interoperability among health institutions with no prohibitive technical limitations in performance or storage allocation.

Encryption, calculation, and decryption occur at different times based on how the data flow works in the proposed model. Thus, we analyzed the impact of homomorphic encryption for each situation. Encryption is the most time-consuming step and occurs whenever an individual sends data to the DS or when there is a demand for the data of a specific individual from the health consortium. Decryption takes approximately half of the encryption process. This process occurs whenever a node in the health consortium needs to obtain the result from a calculation or receive encrypted data from an individual in an SDV. Calculation tends to be the most frequent operation in the model and is also the best-performing cryptographic step. When calculations are run on high volumes of data, it takes less than 3 min to process more than one million records.

We analyzed the performance and profiling results of Kocabas et al. [29], and our proposed model obtained better results. Many factors influence this difference. In the study conducted by Kocabas et al. [29], BGV is the FHE algorithm, whereas our model uses the BFV algorithm, which is an evolution of the BGV algorithm. The payload in each round in the study conducted by [29] was an array of 200 ECG records, and we computed a binary value v∈[0,1]. Finally, the hardware used in the previous study [29] was a dual-Xeon E5450 node of 16 GB RAM and quad-cores @3GHz, while we used an Apple M1 8 Core of 3.2 MHz and 16 GB RAM.

Regarding security and vulnerability scenarios, our study offers a differentiation from the related studies of Yazdinejad et al. [22] and She et al. [21], where private keys are managed by health institutions and not by individuals. This scenario raises security issues where institutions can generate transactions on behalf of the Revindividual without consent.

## 8. Limitations

This study focuses on applying blockchain and homomorphic encryption to the healthcare sector and introduces new elements to segregate the responsibilities of data management. To accomplish this, we introduce the element of DS, which is responsible for managing PHR outside the institutional environment. The addition of this new element results in certain limitations in the model. As DS is not a traditional element in the health industry; companies need an incentive to provide such services. People must also enroll in a DS to share their data with health institutions. It can generate costs to individuals or a demand to share their data in a data-monetization model.

Our model aims to address potential conflicts of interest regarding PHR and health institutions. Such institutions are already regulated by acts, such as HIPAA and GDPR, and cannot sell or profit directly from data. Thus, the segregation of responsibilities in the proposed model aims to avoid scenarios in which health institutions can act as service providers that maintain the PHR base for individuals and potentially make a profit from such services.

Health monitoring devices and health platforms commonly gather PHR on proprietary systems in a non-standardized format. This scenario requires a specific data-gathering solution for each supported device and platform and impacts the practical adoption of the proposed model in the short term. Another limitation is that the DSs must be part of each consortium to exchange data from the individuals with health institutions. This can result in situations where an individual is required to share their data with a health institution, but the DS is not part of the same consortium as the institution.

Finally, the data scope only considers numeric data in a format that supports homomorphic encryption calculations. It does not consider more complex data formats and standards, such as HL7 FHIR. Data in this format are nevertheless suitable for sharing in an encrypted form but not for calculations.

## 9. Conclusions

This study proposes a distributive model to elevate trust in PHR data. The model focuses on two significant challenges: protecting personal privacy and obtaining trustworthy information on healthcare management. We propose new elements called data wards and shared vaults to segregate responsibilities related to personal data and promote trust mechanics to foster network behavior among participants. With an end-to-end encryption model, it is possible to support the exchange and calculation of information regarding healthcare without exposing individuals owing to the homomorphic encryption technique.

We explored different aspects of the model, such as privacy, performance, and node communication in the blockchain. The benchmarks and profiling of homomorphic encryption algorithms demonstrate the applicability of such techniques. The proposed algorithm calculated over one million records in less than 3 min and allowed sharing of a new PHR entry in less than 30 s, which could support calculations and the publication of pandemic outbreak data in practical applications. Homomorphic encryption provides a set of techniques to support the statistical calculations of encrypted data as a mechanism to protect personal privacy and provide public-interest information regardless of any individual representation of the data.

In the future, standards for data in health devices and semantic interoperability will have more adoption. Thus, the model can explore alternatives, such as tokens, to improve the role of the DS in supporting data monetization as an incentive for individuals and service providers. The model should be tested in real-sized blockchain consortia to evaluate the impact of latency and consensus on the overall performance. This study is a part of a multi-organizational project in Brazil called MinhaSaúdeDigital (MyDigitalHealth in English), and we are implementing this model in more than ten participating institutions.

## Figures and Tables

**Table 1:** Table of acronyms.

Acronym	Definition
DHT	Distributed Hash Table
DS	Data Steward
EHR	Electronic Health Record
FHE	Fully Homomorphic Encryption
GDPR	General Data Protection Policy Regulation
HIPAA	Health Insurance Portability and Accountability Act
IPFS	Interplanetary File System
PHR	Personal Health Record
SDV	Shared Data Vault

**Table 2:** Related work: Comparison analysis of previous studies in terms of opportunity for individuals to create cryptographic keys, digitally agree or deny access to PHR, use their own keys to encrypt data, and choose the infrastructure to store PHR. Comparison of previous studies in terms of their focus on EHR and PHR.

Author	Year	Main Focus	Key Generation	Grant Access	Revoke Access	Encrypt Data	Manage Location
Roehrs et al. [4]	2017	Interoperability	X	-	-	X	X
Sonkamble et al. [37]	2017	Interoperability	-	X	X	-	-
Ghadamyari and Shame [20]	2019	Data Analysis	-	-	-	-	-
She et al. [21]	2019	Privacy	-	-	-	X	-
Radhakrishnan et al. [38]	2019	Security	-	X	-	-	-
Niu et al. [39]	2019	Interoperability	-	X	-	-	-
Yazdinejad et al. [22]	2020	Authentication	-	X	-	-	-
Zhuang et al. [40]	2020	Information Exchange	-	X	-	-	-
Muizz Mahdy [41]	2020	Interoperability	X	X	-	-	-
Misbhauddin et al. [19]	2020	Scalability	X	X	X	-	-
Madine et al. [42]	2020	Interoperability	X	X	-	X	-
Sun et al. [26]	2020	Security	-	-	-	-	-
Ghani et al. [43]	2020	Information Exchange	X	X	X	-	-
Mubashar et al. [32]	2021	Information Exchange	-	-	-	-	-
Wang et al. [44]	2021	Interoperability	X	X	-	X	X
Proposed Model	2022	Interoperability	X	X	X	X	X

**Table 3:** Data location of previous models: Most models use a combination of on-chain and off-chain storage owing to blockchain scalability issues.

Author	Year	Main Focus	On-Chain	Off-Chain Centralized	Off-Chain Decentralized
Roehrs et al. [4]	2017	Interoperability	None	-	Health Record
Sonkamble et al. [37]	2017	Interoperability	Smaller data	Images and reports	-
Ghadamyari and Shame [20]	2019	Data Analysis	Health Record	-	-
She et al. [21]	2019	Privacy	Data Hash	-	-
Radhakrishnan et al. [38]	2019	Security	Health Record	-	-
Niu et al. [39]	2019	Interoperability	Keywords	Health Record	-
Yazdinejad et al. [22]	2020	Authentication	Data Hash	-	-
Zhuang et al. [40]	2020	Information Exchange	Data Hash	Health Record	-
Muizz Mahdy [41]	2020	Interoperability	Health Record	-	-
Misbhauddin et al. [19]	2020	Scalability	Data Hash	-	Health Record
Madine et al. [42]	2020	Interoperability	Metadata, Data Hash	-	Health Record
Sun et al. [26]	2020	Security	Data Hash	-	Health Record
Ghani et al. [43]	2020	Information Exchange	Data Hash	-	Health Record
Mubashar et al. [32]	2021	Information Exchange	N/A	-	Health Record
Wang et al. [44]	2021	Interoperability	Data Hash, Shared Key	-	Health Record
Proposed Model	2022	Interoperability	Metadata, Data Hash	-	Health Record

**Table 4:** Example scenario with actions taken by the participants in the proposed model.

Medical Event	Technical Aspects
Individual becomes a patient at a medical institution to treat a heart disease.	Health institution queries the blockchain and obtains the previous examinations from other institutions in the same consortium already available in an SDV.
Health professionals request access to the PHR: heart rate for the past three months.	Health institution communicates with the DS to request data access; the individual receives a notification and signs the transaction.
Patient allows access for ten days.	The DS creates a shared vault for the health institution: a transaction on the blockchain containing the CID of the requested PHR.
The screening process is conducted.	The health institution generates and stores new medical records of the given patient and refers to their public key.
Data from the health institution are shared with the patient.	Blockchain is updated, and the individual encrypts the data received and sends it to the DS.
The health institution notifies that access to the data is no longer necessary, and that the local PHR copy is discarded.	A transaction is made on the blockchain regarding the existing SDV signed with the private key of the health institution.

**Table 5:** Results summary: results compared to related work.

Aspect	Related Work	Result Comparison
Homomorphic Encryption	Kocabas et al. [53]	Better performance with a higher encryption factor
	She et al. [21]	More homomorphic encryption operations with the use of FHE (BFV) instead of Partially Homomorphic Encryption (Paillier)
Privacy protection scenarios	Ghani et al. [43]	Security improved with the use of data encryption instead of steganography
	Wang et al. [44]	Security improved with the use of asymmetric keys instead of symmetric keys

**Table 6:** Algorithm profiling: parameters *n* and *q* as the polynomial modulus degree and coefficient modulus, respectively, in a BFV encryption based on a previous study [36]. The steps of encryption, addition, and decryption are depicted in the rows.

*n*	*q*	Encryption (ms)	Addition (ms)	Decryption (ms)
1024	27	2.3673	0.0117	2.5798
2048	52	2.3559	0.0117	2.4655
4096	86	2.4351	0.0118	2.6629

**Table 7:** Storage consumption: As the (n,q) pair increases, the cyphertext string size (KB) also increases. Each column *S* corresponds to the average size of the registry in plain, encrypted, and compressed formats, while each column *T* refers to the total storage amount for a set of 100,000 registries.

		Plain Text		Encrypted		Compressed	
***n***	***q***	$S_{\mathrm{plain}}$	$T_{\mathrm{plain}}$	$S_{\mathrm{enc}}$	$T_{\mathrm{enc}}$	$S_{\mathrm{comp}}$	$T_{\mathrm{comp}}$
1024	27	212 B	21 MB	11.5 KB	1.12 GB	8.7 KB	851 MB
2048	52	212 B	21 MB	40.6 KB	3.96 GB	30.7 KB	3 GB
4096	86	212 B	21 MB	71.5 KB	6.98 GB	54.2 KB	5.29 GB

**Table 8:** Security and privacy scenarios.

Indicator	Compromised Data	Data Format	Extension	Mitigation
Exp-1	Single person’s PHR	Encrypted/Plain	Person’s history; new records	Two-factor authentication; Trusted Peers list
Exp-2	Data Stewards off-chain data	Encrypted	A group of persons’ PHR	Not necessary
Exp-3	Medical Institution’s off-chain data	Encrypted/Plain	A group of person’s PHR	Reduced Shared Data Vault duration
Exp-4	Consortium node	Encrypted	Incoming transactions; synchronized ledger	Not necessary

## Data Availability

All data used in the experiments where collected from the Center for Disease Control and Prevention of the United States of America at the following address: https://data.cdc.gov/Case-Surveillance/COVID-19-Case-Surveillance-Public-Use-Data/vbim-akqf—accessed on 15 October 2022.
